# Human Papillomavirus (HPV) Infections Among Participants Undergoing Chlamydia trachomatis Testing in Reunion Island (RUN-SurV-HPV Study): Protocol for a Prevalence Study

**DOI:** 10.2196/47379

**Published:** 2023-10-31

**Authors:** Maxence Gandelin, Phuong Lien Tran, Emmanuel Chirpaz, Marie-Christine Jaffar-Bandjee, Nicolas Traversier, Dorothy Machalek, Antoine Bertolotti

**Affiliations:** 1 Service des Maladies Infectieuses, Dermatologie Centre Hospitalier Universitaire La Réunion Saint-Pierre Réunion; 2 Service de Gynécologie et Obstétrique Centre Hospitalier Universitaire La Réunion Saint-Pierre Réunion; 3 Institut National de la Santé et de la Recherche Médicale Centre d'Investigation Clinique 1410 Centre Hospitalier Universitaire La Réunion Saint-Pierre Réunion; 4 Registre des cancers de La Réunion Centre Hospitalier Universitaire de La Réunion Saint-Denis Réunion; 5 Laboratoire de Microbiologie Centre Hospitalier Universitaire La Réunion Saint Denis Réunion; 6 The Kirby Institute University of New South Wales Sydney Australia; 7 Centre for Women's Infectious Diseases The Royal Women's Hospital Melbourne Australia

**Keywords:** human papillomavirus, HPV, Reunion Island, epidemiological study, vaccination, vaccine, sexual health, sexual transmission, sexually transmitted infection, STI, epidemiological, epidemiology, cross-sectional, genotyping

## Abstract

**Background:**

Infection by human papillomavirus (HPV) induces various cancers, mainly cervical cancer but also anal and pharyngeal cancers. Reunion Island is a French department in the Indian Ocean. Due to the history of its settlement, the island’s population has a wide variety of origins, and the population’s sociodemographic characteristics differ from those of the French mainland. These characteristics make the island’s population an original French population in its own right, particularly in terms of health. Cervical cancer incidence and mortality in Reunion Island are 10.4 per 100,000 and 4.4 per 100,000, respectively, both of which are much higher than those in mainland France. There is also a disparity in the prevalence of different HPV types, with types 33 and 52 being overrepresented and type 18 being underrepresented. However, vaccination and screening coverage in Reunion Island is low. It is important to understand the burden of infection and its risk factors in members of the young Reunionese population at risk of HPV infection to promote and evaluate the implementation of future vaccination and screening programs on a larger scale.

**Objective:**

The RUN-SurV-HPV study will have the following four objectives: (1) to describe the prevalence of HPV genotypes in a population at risk of sexually transmitted infections in Reunion Island; (2) to describe the prevalence of HPV genotypes by anatomical site, gender, and sexuality; (3) to describe the correlates and risk factors for HPV detection; and (4) to examine HPV genotypes between different anatomical sites.

**Methods:**

Cross-sectional analyses of 1200 routine vaginal, anal, pharyngeal, and urinary samples collected between October 2020 to December 2022 from female and male patients aged 16 to 30 years undergoing *Chlamydia trachomatis* testing at a sexually transmitted infection (STI) testing center at Reunion Island will be performed. The population included 333 women who all had vaginal and pharyngeal swabs, with 80 of them also having had an anal swab. There are 167 men who have sex with men who have had anal and pharyngeal swabs, and 120 men who have sex with women who have had a urine swab only. Results will be correlated with sociodemographic and clinical data collected routinely during the consultations. HPV detection and genotyping will be performed using the Anyplex II HPV28 detection assay (Seegene).

**Results:**

The first polymerase chain reactions should begin in November 2023. The first results should be submitted for publication in 2024.

**Conclusions:**

The study will determine HPV prevalence by age, sex, male sexual preference, human immunodeficiency virus status, and STI co-infection. Collecting data from young patients not usually routinely screened for HPV infection will be a simple and reproducible way of better understanding local specificities, encouraging vaccination campaigns in the short-term, and evaluating their effectiveness in the future.

**International Registered Report Identifier (IRRID):**

DERR1-10.2196/47379

## Introduction

Human papillomavirus (HPV) is the most common sexually transmitted infection (STI) in the world. There are more than 100 types of HPV, with 13 classified as oncogenic. Of these, HPV types 16 and 18 are the cause of 70%-80% of invasive cervical cancers [1-3], which comprise the fourth most common cancer worldwide [4]. HPV infections also cause cancers of the vulva, vagina, penis, anal canal, and oropharynx; therefore, they affect both women and men.

Reunion Island is a French overseas department located in the Indian Ocean that is populated by more than 850,000 inhabitants. Due to the history of its settlement, the population of the island has very varied origins (ie, East Africa, the Comoros Islands, Madagascar, India, China, and Europe) that differ from mainland France. The geographical distance and its sociodemographic characteristics make it an original French population in its own right. In Reunion Island, cervical cancer is the fourth most common type of cancer in women. From 2013 to 2015, cervical cancer incidence and mortality were 10.4 per 100,000 and 4.4 per 100,000, respectively, making it the eighth deadliest cancer, 2 times higher than in mainland France [5]. These disparities can be explained partly by a low cervical cancer screening rate in the 1990s and 2000s [6] compared to the French national average, but the trend now seems to be reversing. Cervical cancer screening is a secondary prevention approach that is universally considered to be effective in reducing the incidence and mortality of this cancer in the general population. It is recommended in most developed countries. Furthermore, several studies have shown that risk factors for infection (eg, early sexual activity, high parity, precariousness) by the various STIs do not totally overlap with those in mainland France, and are, in fact, overrepresented in Reunion Island [7,8]. However, the only study to examine the link between this overrepresentation of risk factors and the incidence of cervical cancer in Reunion Island did not explain this difference in cancer incidence rates [9].

The first HPV vaccine was marketed worldwide in 2006. Initially, the first 2 vaccines protected against 2 genotypes (16 and 18) or 4 genotypes (6, 11, 16, and 18). Currently, the latest vaccine protects against 9 genotypes (6, 11, 16, 18, 31, 33, 45, 52, and 58). In 2007, the French High Authority for Health (HAS: Haute Autorite de la Sante) first implemented a community-based vaccination program that recommended HPV vaccination for girls aged 11 to 14 years with the possibility of catching up until 19 years of age. In 2019, the program was extended to include boys in the same age groups [10]. This officially came into effect in January 2021 [11]. In 2021, vaccination coverage was only 37.4% in mainland France [12]; it thus remains one of the lowest in Europe [13]. In Reunion Island, coverage was only 12.2% in 2021 based on the results of one available local research study [12]. Coverage is far short of the 90% elimination target [4]. This low vaccination rate in Reunion Island has in large part been driven by a lack of information and mistrust of vaccination from parents and caregivers [14].

The peculiarity of this population regarding its low vaccination coverage, high cancer rate compared to the national average, diverse ethnic origins, and risk factors encourages local exploration and monitoring of HPV genotype specificity. Furthermore, a previous study in women with atypical squamous cells of undetermined significance (ASCUS) Papanicolaou test results (age: mean 35.4, SD 12.3 years) and cervical biopsies of high-grade histological lesions or cancer (age: mean 48.6, SD 17.1 years) identified that the distributions of different types of HPV in Reunion Island were not similar to those observed in mainland France (eg, underrepresentation of HPV 18, overrepresentation of HPV 52, high proportion of co-infections) [15]. Moreover, there are no data on the prevalence of oncogenic HPV in Reunion Island in women younger than 30 years (for the cervical, pharyngeal, and anal areas), men who have sex with women (MSW), or men who have sex with men (MSM).

In the RUN-SurV-HPV study, we want to analyze the distribution of different oncogenic HPV types (16, 18, 26, 31, 33, 35, 39, 45, 51, 52, 53, 56, 58, 59, 66, 68, 69, 73, and 82) in a population of women and men at risk of STIs attending an STI testing center for *Chlamydia trachomatis* (CT). The inclusion of young patients at risk of STIs, and therefore at risk of HPV infection, including both women and men, will enable us to study a population in which the incidence of HPV infection will likely be high. The inclusion of men, whether MSM or MSW, is in line with the recent extension of vaccination to French men and women, regardless of their sexuality [10]. Indeed, MSM have a higher risk of anal cancer [16], and MSW, even if in a smaller proportion, are still affected by HPV-induced cancers and can be vectors of infection for women. By including men, this study will improve our understanding of HPV infection in men and provide arguments to encourage them to be vaccinated. This type of study will allow us to obtain a more precise mapping of different oncogenic HPV genotypes in this population and evaluate an early surveillance system for changes in HPV distribution following vaccination campaigns.

The main objective is to describe the prevalence of infection by at least 1 of the 7 high-risk oncogenic HPV genotypes contained in the GARDASIL 9 HPV vaccine (16, 18, 31, 33, 45, 52 and 58) in women younger than 30 years who have had a vaginal swab and MSM who have had an anal swab for CT infection in the Reunion Island population.

The secondary objectives are as follows:

1. To describe the prevalence of infections by different high-risk (16, 18, 26, 31, 33, 35, 39, 45, 51, 52, 53, 56, 58, 59, 66, 68, 69, 73, and 82) and low-risk (6, 11, 40, 42, 43, 44, 54, 61, and 70) HPV genotypes in samples from routine vaginal, anal, or pharyngeal CT testing from women younger than 30 years; urine samples from MSW younger than 30 years; and anal samples associated with pharyngeal CT testing from MSM.
2. To investigate HPV distribution in the same patient (women and MSM) according to the swabbed location.
3. To describe the correlates and risk factors for HPV infection, including age, CT status, status of other STI infections, socioeconomic status, sexuality, reasons for consultation, HPV vaccination status, and human immunodeficiency virus (HIV) status, by patient and type of specimen.

## Methods

### Study Design

This protocol is for a single-center, descriptive, cross-sectional study of all samples collected from male and female patients undergoing routine CT testing at the STI testing center of University Hospital (UH) in south Reunion Island between October 2020 and December 2022.

### Setting

The national recommendation for CT screening in France suggests screening all woman from 15 to 25 years of age (pregnant women included) as well as those older than 25 years with risk factors and all men (all ages). In this study, samples were obtained from patients who went to an STI testing center mainly because of a recent sexual risk or symptomatic genital affection or to be able to remove the condom in a stable relationship.

### Study Population

#### Inclusion Criteria

The following patients were included in the study:

1. Those who consulted the STI testing center of the UH site in south Reunion Island between October 1, 2020, and December 31, 2022;
2. Female patients younger than 30 years because the national recommendation for HPV screening is from 30 years of age and following a younger population would provide insight about the first impact of the national vaccination program;
3. MSM (all ages) because they have a higher risk of anal cancer;
4. MSW younger than 30 years because they are also affected by HPV-induced cancers, are included in the new French vaccination recommendations, and comprise a specific population that is able to infect women;
5. Those who were tested for CT infection on at least 2 different anatomical sites (vaginal, pharyngeal, and anal) for women, first pass urine for MSW, and at least 2 different sites (anal and pharyngeal) for MSM;
6. Those whose residual samples were available at the microbiology laboratory of the UH of Reunion Island.

#### Exclusion Criteria

The following patients were excluded from the study:

1. Those who were outside the study age range,
2. Those who did not consent to the use of their data for research,
3. MSW with no sites collected and women and MSM with less than 2 sites collected,
4. Those for whom there was insufficient biological material.

### Data Collection

Social and demographic data were extracted from routine clinical records. These data were collected for each person presenting for screening via a self-administered questionnaire that was routinely administered and completed during the consultation (Multimedia Appendix 1). This questionnaire met the annual variables requested by France Public Health (Sante Publique France) from STI testing centers. It included age, gender, sample type, CT test result, HPV vaccination status (self-reported), male sexual preference, and social and demographic data. The collected information was anonymous in this testing center, but patients had a choice to link their clinical data with an anonymity number or their name for each sample. For this research, data will be entered into a secure anonymous database according to current national guidelines.

### Data Extraction

#### Data Processing by the Laboratory

Sample collection at the laboratory, the supply of sampling equipment, and the transport of the samples was carried out in compliance with the NF EN ISO 15189 standard, thus guaranteeing the integrity of the samples and their traceability.

Samples were transported to the laboratory at 4 °C in eSwab transport media (COPAN Diagnostics) for swabs (vaginal, oral, and anal) and in sterile preservative-free flasks for urine. After the CT polymerase chain reactions (PCRs) were performed, residual samples were frozen at –80 °C to perform this study.

Enclosures equipped with range temperature sensors (SpyRF) are metrologically monitored using a monitoring web platform software (JRI-MySirius), allowing the exploitation of alarms and data. An Ethernet modem is installed in each lab, thus ensuring radio frequency communication with the SpyRF.

#### HPV Detection and Genotyping

Samples will be tested with the Anyplex TM II HP28 Detection kit (Seegene), a semi-qualitative test for the detection of DNA and genotyping of HPV types. DNA extraction will be performed using the STARMag Universal extraction kit (Eurobio Scientific) on the STARlet (Seegene) automated system. After extraction, genotyping will be done by amplification on a CFX96 TM thermocycler (BioRad). This kit uses the human housekeeping gene, beta globin, as an endogenous internal control to verify the DNA extraction, PCR reaction, and presence of cells in each sample. This real-time multiplex assay allows simultaneous amplification, detection, and differentiation of target nucleic acids of 28 HPV types (6, 11, 16, 18, 26, 31, 33, 35, 39, 40, 42, 43, 44, 45, 51, 52, 53, 54, 56, 58, 59, 61, 66, 68, 69, 70, 73, and 82).

#### Processing of Medical Records, Questionnaires, and Patients’ Results

Using standard software extraction tools, throughout the study, researchers will generate a list of all the information included in patients’ records and self-administered questionnaires issued and completed on the day of collection. This anonymous information will then be extracted into a secure Excel spreadsheet (Microsoft Corp). HPV genotyping results will also be extracted into a secure Excel spreadsheet.

### Statistical Considerations

#### Sample Size

Sample collection started in October 2020 and ended in December 2022. For women, 1436 swabs were collected from 665 women. A vaginal and pharyngeal swab was collected on the same day from 333 women younger than 30 years, 80 of whom also had an anal swab. For MSM, 738 samples were collected from 258 MSM. An anal and pharyngeal swab was collected on the same day from 167 MSM. Finally, 661 MSW provided a urine sample and 324 were younger than 30 years. Between 120-180 urine samples from MSW will be randomly selected for inclusion in the study, depending on the availability of stored samples from women and MSM. A total of 1200 samples will be tested (Table 1). Due to the absence of data with this type of methodology in Reunion Island, an estimated 95% CI for different prevalence levels was calculated using STATA 17.0 software (StataCorp) from vaginal swabs from women and anal swabs from MSM to address our primary objective (Table 2). This ranking was from 1% to 50% prevalence of HPV infection corresponding to 1 of the 7 potential oncogenic HPV genotypes contained in GARDASIL 9 in our previous study on ASCUS Papanicolaou tests and cervical biopsies (conservative assumption) [15].

**Table 1:** Description of the number of samples by gender, sexuality, and anatomical site of collection obtained between October 2020 and December 2022 from patients of all ages undergoing *Chlamydia trachomatis* testing at a sexually transmitted infection testing center in Reunion Island as part of the RUN-SurV-HPV study.

Anatomical site (n=1200)	Women (n=333), n	MSM^a^ (n=167), n	MSW^b^ (n=120), n
Pharyngeal	333	167	N/A^c^
Vaginal	333	N/A	N/A
Anal	80	167	N/A
Urine	N/A	N/A	120

^a^MSM: men who have sex with men.

^b^MSW: men who have sex with women.

^c^N/A: not applicable.

**Table 2:** Estimation of the 95% CI for human papillomavirus prevalence among women and men who have sex with men (MSM) who visited a sexually transmitted infection testing center from October 2020 to December 2022 in Reunion Island as part of the RUN-SurV-HPV study.

Prevalence (%)	Women (n=333), 95% CI	MSM (n=167), 95% CI
1	0.30-3.30	0.10-4.25
5	3.00-8.00	2.09-9.22
10	6.90-13.60	6.04-15.79
20	15.90-24.80	14.00-26.61
30	25.15-35.26	23.10-37.49
40	34.63-45.42	32.62-47.97
50	44.35-55.35	41.88-57.52

#### Analysis Plan

Patient characteristics will be described in terms of numbers and percentages for qualitative variables and in terms of mean and SD, median and IQR, and range for quantitative variables. The prevalence of HPV infections will be expressed as percentages. The exact binomial method will be used to calculate 95% CIs. Analysis of primary assessment criteria will be performed per patient considering only vaginal samples for women and anal samples for MSM. For analysis of factors associated with HPV infections, associations with factors of interest will be carried out for qualitative variables by chi-square test or Fisher exact test (depending on the conditions of validity); comparisons concerning continuous variables will be carried out by Student *t* test or Wilcoxon test depending on the conditions of validity. To account for confounding effects and to identify factors independently associated with HPV infections, a multivariate analysis by logistic regression will be performed. The dependent variable will be HPV infection (yes or no). The independent variables will come from the self-administered questionnaire and laboratory results and will include sex, age, CT* *status, status of other STIs, socioeconomic status, sexuality, reasons for consultation, HPV vaccination status, and HIV status. The independent variables entered in the model will be variables for which the significance level will be *P*<.2 in the bivariate analysis; variables will be selected using backward stepwise regression with a significance level for removal from the model of *P≥*.1. To investigate whether factors associated with HPV infection differ according to sex and type of specimen, subgroup analyses will be performed by swab site and sex. McNemar tests will be used to assess differences between matched samples. Cohen κ will be calculated to check the concordance of HPV infections between matched samples from the same patient. To assess this concordance for women and MSM, individuals with samples from 3 sites will be preferred over those with samples from only 2 sites. A random selection will be made to select individuals with only 2 samples in order to meet the allowable sample size.

The significance threshold for the tests will be 5% *(P<*.05). CIs will be calculated at 95%. The statistics will be calculated with the STATA 17.0 software.

### Ethical Considerations

The expected benefits outweigh the potential risks to patients.

National cervical cancer screening with HPV PCR is recommended by the HAS for individuals 30 years and older. In our study population, and in accordance with national recommendations, there is no obligation to follow up or to carry out additional examinations in the event of a positive result for HPV at the cervix-vaginal level, particularly if it is one of the oncogenic or probably oncogenic HPV types. The natural history of HPV infections is specific to the population younger than 30 years. The incidence and prevalence of HPV infections are at their highest levels due to the presence of major risk factors, such as number and frequency of new sexual partners. These infections are responsible for minor cytological lesions that evolve spontaneously and favorably in the majority of cases. Indeed, in this population, viral clearance is earlier and more marked than in older women, with 50% clearance of the infection within the first 6 months and 90% clearance within 2 years. In France, to date, there is no recommendation for HPV screening, nor is there a recommendation for treatment in the event of the discovery of an oncogenic HPV infection, whether it be anal, pharyngeal, or urinary, in women, MSM, or MSW.

An information note will be given at the time of the consultation setting out the objectives of the research organized based on data collected during care.

In compliance with national legislation, ethics approval has been obtained from the ethics evaluation committee of INSERM (French National Institute of Health and Medical Research; Opinion number 22-953) and all procedures were performed in accordance with the ethical standards of the responsible committee on human experimentation and with the Helsinki Declaration of 1975.

## Results

Patient recruitment began in October 2022 and ended in December 2022. Approximately 1200 samples were collected from 333 women, 167 MSM, and 120 MSW. Funding was obtained thanks to the support of the University Hospital of La Reunion. The first PCRs are scheduled for November 2023. We expect to submit the first results in 2024.

## Discussion

### Expected Outcomes

The results of this study, carried out in a largely unvaccinated population, will enable us to give more details on the distribution of HPV infections in the Reunion Island population before vaccination. Currently, in France, but also locally in Reunion Island, there is a desire to promote a community-based vaccination program with GARDASIL 9 to achieve a national coverage target of 80% among those aged 11 to 19 by 2030 [17,18]. The identification of the transmission of genotypes that are mostly covered by the 9-valence vaccination of patients in Reunion Island will help to reassure the general population and the doctors about the necessity of this vaccination. For MSM, it will be the first study to assess the prevalence of HPV in anal samples.

Vaccination has already been shown in several studies to be effective in reducing HPV infections [19] and precancerous lesions [20]. The Swedish study by Lei et al [21] followed a cohort of more than 1.6 million patients aged 10 to 30 years between 2006 and 2017 before, during, and after large-scale vaccination was introduced in Sweden. It showed a significant reduction in cervical cancer in vaccinated populations, with a favorable incidence rate ratio of 0.37 (95% CI 0.21-0.57) after covariate adjustment.

The Swedish team of Söderlund-Strand et al [22] has already used residual CT screening samples to carry out a prevalence study of HPV infections in a defined Swedish region and population of women and men in 2008. From 2012 to 2013, the team carried out the same study on the same population and in the same region of Sweden to identify changes in the prevalence of HPV infections. It is interesting to note that the first state-funded subsidies for HPV vaccination in Sweden were introduced in 2006 and the state vaccination campaigns began in 2012. In both cases, these vaccination recommendations were aimed solely at girls. Vaccination coverage was measured using various national registers. In 2008, when the first study was carried out, vaccination coverage among women in the population studied was 1.2%. Between 2012 and 2013, vaccination coverage among women was between 6.6%-7.5%. Coverage was highest among women and girls younger than 23 years, with a maximum of 77.8% coverage among 14-year-olds. In this study, the majority of samples were genital and came from women aged 18 to 23 years. This study identified a significant drop in the prevalence of HPV genotypes 6, 11, 16, 18, 52, and 56 in women of all ages between 2008 and 2012-2013. These results are difficult to interpret given that detection of HPV by PCR in urine is known to be low. There was variability in the prevalence of HPV genotypes not covered by the vaccine, with an increase in HPV genotypes 52 and 56 but a decrease in genotype 31. The fact that the prevalence of HPV genotypes covered by the vaccine decreased mainly in the age groups of the most highly vaccinated populations is a strong argument for vaccine efficacy and is consistent with studies carried out in other countries. On the other hand, the decrease in the prevalence of HPV infections by covered genotypes in the population studied and the fact that certain noncovered genotypes tended to increase is not consistent with the data on cross-protection by the vaccine. It would therefore be interesting to monitor locally for the possible emergence of these HPV genotypes [22]. Other teams have carried out repeated cross-sectional surveys to measure vaccine pressure on HPV infections; for example, Markowitz et al [19] carried out 2 studies in the prevaccination era (2003-2006) and vaccination era (2007-2010) among more than 4000 patients recruited through national public health surveys, which included cervico-vaginal HPV tests in women aged 14 to 59 years. This study showed a significant drop in the prevalence of HPV 6, 11, 16, and 18 vaccines in women aged 14 to 19.

These different studies, each with their own specific design, demonstrate the value of carrying out this type of cross-sectional study repeatedly over time. With regard to Reunion Island and the prospect of repeating the RUN-SurV-HPV study in several years’ time, it will be important to monitor changes in vaccination coverage locally. The next study should be carried out sufficiently far in advance of the current one so that vaccination has had time to become more widespread in Reunion’s population. We can then hope to demonstrate the effect of vaccination with GARDASIL 9 by identifying a significant reduction in HPV infections and monitor the appearance of other genotypes in our population of women and MSM younger than 30 years.

### Comparison With Previous Work

In this study, we expect to have different results from the studies already conducted in Reunion Island. The last genotyping study of HPV infections in Reunion Island was conducted from 401 ASCUS Papanicolaou tests and 94 cervical biopsies with histopathological diagnosis of high-grade lesions or cancer performed between 2008 and 2012 [15]. After analysis, 162 (40.4%) Papanicolaou tests and 63 (67%) biopsies were HPV DNA positive. Of the 162 Papanicolaou tests, HPV genotyping was possible for 100 samples. Co-infection was found in 60% of the Papanicolaou tests. The most common HPV genotypes in the Papanicolaou tests were 51 (12.3%), 31 (10.3%), and 16 (8.4%). Of the 63 HPV DNA positive biopsies, the most frequent HPV genotypes detected were 16HR (41.3%), 52HR (16.3%), and 33HR (11.5%). Co-infections were detected in 50.7% of these biopsies [15]. It will therefore be interesting to compare our results with those of this study, which is already more than 10 years old, taking into account that the type of sampling and the characteristics of the patients will not be superposable. In addition, in our work, the number of samples taken will be twice as high and will likely have different HPV positivity rates because the samples will be taken in a population largely free of any induced HPV lesions but at risk of STIs. The samples will be more recent in healthy participants for whom social and demographic data will be available. Moreover, the evolution of HPV infection from asymptomatic to precancerous or even cancerous is slow and takes several years, reinforcing the idea that the previous study may no longer reflect the seroprevalence of HPV types in the Reunion Island population. Because of the size of our sample, our data will be more robust than the previous study, but also more up to date. Our results will not be representative of the general population of Reunion but will represent the populations most concerned by vaccination and the prevention of induced HPV lesions.

Identifying the prevalence of HPV genotypes by analyzing samples collected for CT testing has been done in several countries to map HPV infections and assess the impact of their vaccination programs [23,24]. In a pilot Australian study by Shilling et al [23], 362 residual genital CT samples were matched to vaccination status and analyzed for HPV using the Anyplex II HPV 28 detection assay. All samples were from young women aged 16 to 24 years, the majority of which were of cervical origin. In the samples analyzed, 7.8% were positive for CT. HPV genotypes 16 or 18 were detected in 3% of the women, while HPV genotypes 31, 33, 45, 52, or 58 were detected in 19% [23]. The Swedish study by Söderlund-Strand et al [24] analyzed more than 44,000 residual CT specimens, mostly from women. In women, HPV test positivity was 37.8%, of which 10% were positive for HPV 16 and 6% for HPV 51. In men, 11.2% of the samples collected were positive for HPV, primarily HPV 16 (2.1%) and HPV 6 or 51 (1.7%) [24]. In these two studies, the high prevalence of HPV infection in populations consulting for CT testing provides a suitable sample for surveillance surveys of HPV vaccination programs. These previous studies from different countries reinforce the feasibility and relevance of our study.

The concordance of HPV genotypes in the same individual at different anatomical sites remains controversial in the literature. Müller et al [25] identified a much higher prevalence of anal HPV than pharyngeal and urinary HPV in 200 MSM but found concordance for HPV 16. The work of Nugent et al [26] identified good concordance between HPV-specific anal swabbing and residual swab analysis but the latter was a combination of pharyngeal, urinary, and anal swab residue. Nakashima et al [27], on the other hand, identified good concordance between HPV in the pharyngeal and urinary tracts. The British study by King et al [28] did not identify a concordance between HPV detected at the anal and pharyngeal levels in HIV-negative MSM. Finally, the Vietnamese team of Le et al [29] identified a weak concordance of HPV infections detected at the pharyngeal and genital levels in men co-infected with other STIs. Indeed, out of 198 patients analyzed, HPV was detected at both sites with only 1 genotype match in 1 patient. All these very discordant data from the literature lead us to explore the specificities of the Reunion Island territory locally to better understand the modalities of HPV infection of different anatomical sites in the same person.

### Strengths of the Study

The strengths of this study lie in the originality of its design, with a data collection method (anonymous and tube-based analyses) that is not restrictive for the participants and investigators. This method makes it possible to include a large number of participants for more representative results. It is also easily reproducible over time, which will make it possible to repeat this type of study to monitor the evolution of HPV infections in the Reunion Island population.

The characteristics of the study population are also interesting. First, it is composed of both women and men, which coincides with the recent extension of French vaccination recommendations against HPV to adolescents of both sexes [10]. To our knowledge, there has been no previous study carried out in Reunion Island on the prevalence of HPV in men and in the pharyngeal or anal region in women. The young age of the participants matches the period of life when the prevalence and incidence of HPV infection is highest. Patients consulting STI testing centers are often in a precarious situation with risk behaviors that favor co-infections with another sexually transmitted disease, such as risk factors for HPV infections. These characteristics will therefore give us a better idea of the current mapping of HPV infections in Reunion Island. All these social and demographic data will be easily accessible to us thanks to the self-administered questionnaires systematically filled in during each consultation. These data will allow us, through subgroup analyses, to identify in our population the patients most at risk of being infected by an oncogenic HPV genotype, and thus to better target them for future preventative actions. Finally, comparison of the results from different sites in the same patient correlated with sociodemographic data will provide us with additional information on the modes of transmission and infection of HPV and will also allow us to better delimit the complementarity or similarity of screening for this infection to continue or stop it. These results will also provide information on potential targets for HPV vaccination depending on the infected site.

### Limitations of the Study

This study has several limitations. The main limitation is the selection bias of our population. The population consulting STI testing centers is a specific population that cannot be extrapolated to the general population of Reunion Island. This population has an increased risk of HPV infection, and our results may be affected by this. The fact that the patients were recruited via a free screening center where they could consult spontaneously and anonymously reduced the risk of selection bias in our population. In our study, the proportion of under-age patients is likely to be low, even though this population is the main target for vaccination. Unfortunately, despite our efforts, it is difficult to have them consulted spontaneously and to include them while respecting both the anonymity of the consultation and the legislative framework for protecting the data of minors. Finally, this study was only carried out in one screening center, which could affect the results. The results may also be affected by the way the test is performed and its performance, even though it has already been validated in several studies [30,31]. The Anyplex II HPV28 detection assay is also one of the tests that will be evaluated by the VALGENT4 study with the aim of obtaining international clinical validation for uterine cervix cancer screening [32]. Although we have taken all possible precautions to avoid technical errors, results may also be influenced by the quantity and quality of viral material available in the samples collected, which is dependent on how the samples are collected, transported, and stored.

### Conclusion

This original epidemiological study, which has already proved its worth in other countries, will have rapid implications by promoting vaccination campaigns and identifying the populations most at risk of being infected by an oncogenic HPV genotype. By comparing the prevalence of HPV infection in the same person and at different anatomical sites, this study will also provide a better understanding of its pathogenicity and mode of transmission and better delineate the complementarity of certain screenings. In the long term, these studies will allow for monitoring of the evolution of HPV infections and the impact of HPV vaccination campaigns in the Reunion Island population.

## Appendix

Multimedia Appendix 1 Santé Publique France data collection questionnaire. Identifying data, such as full date of birth (only year of birth and age will be extracted) and transgender sex (only original sex will be extracted), will not be extracted.

## Abbreviations

ASCUS: atypical squamous cells of undetermined significance
CT: Chlamydia trachomatis
HAS: Haute Autorite de la Sante (High Authority of Health)
HIV: human immunodeficiency virus
HPV: human papillomavirus
MSW: men who have sex with women
MSM: men who have sex with men
PCR: polymerase chain reaction
STI: sexually transmitted infection
UH: University Hospital

## Data Availability

The data sets generated and analyzed during the current study are not publicly available due to privacy issues of the participants. Data are available from the corresponding author request.
