# Supplementary material for: Human Papillomavirus (HPV) Infections Among Participants Undergoing Chlamydia trachomatis Testing in Reunion Island (RUN-SurV-HPV Study): Protocol for a Prevalence Study
Source: JMIR Res Protoc. 2023 Oct 31;12:e47379. doi: 10.2196/47379 (PMC10646676; doi:10.2196/47379)
Supplement: Multimedia Appendix 1 [file resprot_v12i1e47379_app1.docx]

**A – General Information**
**1. Gender:**
  Male (M)
  Female (F)
  Transgender – If yes, specify if transgender M to F or F to M

**2. Date of birth:** a multiple-choice answer (drop down menu)

**3. Country of birth:**

 Metropolitan France

 France Overseas – If yes, specify the department

 Abroad – If yes, specify the country and the time of arrival in France

**4. Department of residence:** a multiple-choice answer (drop-down menu)

**5. Profession:**

 Farmers

 Craftsmen and tradesmen

 Managers

 Intermediate professions

 Employees

 Workers

 No profession

 Students / High school students

**6. Currently working?**
  No, I am not
  Yes, I am

**7. Health Coverage:**

 Health insurance

 Insurance + mutual insurance or ACS (Aide Complémentaire Santé)

 CMU (Universal Medical Coverage)

 CMU-c (Complementary universal medical coverage)

 AME (State medical aid)

 Other

 No health coverage

**B – Your sexuality**

**1. During your life, what types of sexual relationships have you had?**

 Intercourse with men

 Intercourse with women

 Intercourse with people of both sexes

 Never had sex

 I do not wish to answer

**2. In the past 12 months, what types of sex have you had?**
  Sex with men
  Sex with women
  Sex with both sexes
  Never had sex
  I do not wish to answer

**3. In the past 12 months, how many sexual relationships have you had?**

 Male: number

 Female: number

 Transgender: number

**4. In the past 12 months, condom use:**

- With a REGULAR partner:
- For vaginal sex:  NEVER/SOMETIMES -  ALWAYS -  NOT RELEVANT
- For oral sex (fellatio):  NEVER/SOMETIMES -  ALWAYS -  NOT RELEVANT
- With OCCASIONAL intercourse:
- For vaginal intercourse:  NEVER/SOMETIMES -  ALWAYS–- NOT RELEVANT
- For oral sex (fellatio):  NEVER/SOMETIMES -  ALWAYS -  NOT RELEVANT
- During these practices:
- For anal sex (sodomy):  NEVER/SOMETIMES -  ALWAYS -  NOT RELEVANT
- When using sexual objects:  NEVER/SOMETIMES -  ALWAYS -  NOT RELEVANT

**5. Places to meet casual partner(s):**

 Mobile application (Grindr, Tinder, ...)

 Swingers clubs

 Sauna / steam room / ...

 Backroom / sex clubs

 Internet dating site

 Professional network

**6. Do you have sex in exchange for money, drugs, etc.?**

 No

 Yes

**7. Have you been sexually assaulted?**

 No

 Yes

**8. Have you had unprotected sex since your last screening?**

 No

 Yes – If yes, when was the last time you had protected sex?

**C – HISTORY**

**1. HIV status: have you ever been tested for HIV (AIDS) in the past?**

 No

 Yes – If yes, when was the last test? What type of test was it? What was the result of the test?

**2. Other STI (Sexual transmitted infection) status: Have you ever had a sexually transmitted infection? If yes, specify the date.**

 Syphilis

 Gonorrhea

 Chlamydia

 Herpes

 LGV–- lymphogranulomatosis venereum

 Condyloma–- Genital warts

 None of the above

**3. Other STI status: Have you had hepatitis C?**

 Cured

 Chronically carried

 Not cured

**4. Other STI status: Have you been vaccinated against hepatitis B?**

 I don't know

 No

 Yes – If yes, is your vaccination complete?

**5. Are you vaccinated against Human Papilloma Virus (HPV)? CERVARIX ® vaccine, GARDASIL ® vaccine against cervical cancer.**

 I don't know

 No

 Yes – If yes, is your vaccination complete?

**6. Risk Exposures: Have you ever experienced any of the following?**

 Intravenous drug use (syringe)

 Nasal drug use

 Tattooing, piercing, acupuncture, mesotherapy

 Hospitalization abroad

 Transfusion before 1992 (including abroad)

 No exposure on the list

 Other blood exposure (e.g. occupational exposure accident): open answer

**7. Risk exposures: do you have any other risks?**

 Living with someone with hepatitis B

 Living with someone with hepatitis C

 Lived for 3+ months in Africa, Asia, Eastern Europe, Middle East, South America

 Other risk(s)

**D – Reason for consultation**

**1. Do you have one or more of the following clinical signs?**

 Burning of the urine

 Vaginal discharge (white/green)

 Pimples on the body

 Pimple/ulceration on the anus

 Fever/sweats

 White discharge from sex

 Pimple(s)/ulceration on the sex

 Pain in the anus

 Jaundice

**2. Reasons for consultation:**

 Screening without reported risk-taking

 Screening related to risk-taking

 Request for diagnosis because of signs suggestive of HIV / STI

 Rapid test check (TROD)

 Control of a self-test

 Control of a previous positive test (excluding TROD and self-test)

 Treatment of an STI

 Management of an AEV/AES (Accident Exposing to a Viral Risk/Blood Exposure Accident)

 Follow-up of an AEV

 Prescription of PrEP (Pre-exposure prophylaxis)

 Follow-up of a PrEP

 Emergency contraception

 Regular contraception

 Abortion (Voluntary interruption of pregnancy)

 Pregnancy test

 Personalized counselling, informations

 Sexology

 Violence

 Vaccination

 Other

**3. Do you wish to remain anonymous for the treatment of your file?**

**You are anonymous to the medical team for administrative purposes and your results. Please note that your answers to this questionnaire are recorded anonymously.**

 No

 Yes
